# Editorial: Road trip from mild to severe asthmatic inflammation: the traffic lights of biomarkers in asthma management

**DOI:** 10.3389/fmed.2023.1337283

**Published:** 2023-11-30

**Authors:** Konstantinos Porpodis, Ioanna Tsiouprou, Paschalis Steiropoulos, Spyridon Gougousis, Harissios Vliagoftis, Kalliopi Domvri

**Affiliations:** ^1^Pulmonary Department, Medical School, George Papanikolaou Hospital, Aristotle University of Thessaloniki, Thessaloniki, Greece; ^2^Department of Respiratory Medicine, Medical School, Democritus University of Thrace, University General Hospital Dragana, Alexandroupolis, Greece; ^3^Department of Ear Nose Throat, General Hospital of Thessaloniki “G. Papanikolaou”, Thessaloniki, Greece; ^4^Division of Pulmonary Medicine, Alberta Respiratory Centre, University of Alberta, Edmonton, AB, Canada; ^5^Laboratory of Histology-Embryology, Medical School, Aristotle University of Thessaloniki, Thessaloniki, Greece; ^6^Laboratory of Pathology, General Hospital of Thessaloniki “G. Papanikolaou”, Thessaloniki, Greece

**Keywords:** asthma, biomarkers, biologics, personalized treatment approaches, airway remodeling

There is still an unmet clinical need to delineate complex endotypes of asthma and to identify novel biomarkers with high predictive and prognostic value to achieve an optimal personalized approach. This Research Topic, describes current topics in asthma biomarker research, providing a better understanding of the currently available biomarkers, and suggesting new approaches to use these biomarkers in everyday clinical practice for optimal management of patients with asthma.

Our issue assembles several high-quality articles, describing diverse approaches toward asthma management. The eight articles published include four reviews, two mini-review articles, one research article and one opinion article.

Within this context, in a review article, Fouka et al. explored the role of the most important routinely used biomarkers in severe asthma management. The authors presented an overview of the characteristics of biomarkers related to Type 2 (Th2) (eosinophils, FeNO, IgE, periostin) and non-Th2 inflammation, as well as biomarkers identified by proteomic and genomic-based approaches. The authors highlight the value of combinations of different biomarkers to develop algorithms for the initial choice of biologics and the monitoring of the patients' response to treatment.

Along the same topic, Guida et al. demonstrated different aspects of the role of the main Th2 inflammatory biomarkers used in daily clinical practice, applying a critical view of their use. Based on several studies, the authors described distinct blood and sputum eosinophil as well as FeNO cut-offs as predictors of eosinophilic inflammation and reviewed the association between sputum inflammatory phenotypes, FeNO, and clinical outcomes. The authors emphasize that all biomarkers analyzed in this review show high variability over time; however, use of each biomarker within appropriate clinical setting could overcome their limitations and increase the value of their use.

In a slightly different approach, Adatia and Vliagoftis provided a comprehensive overview of the state of current severe asthma biomarkers (blood eosinophils, sputum eosinophils, total IgE, and FeNO) and identified specific phenotypes that are routinely used to predict the efficacy of biologics in severe asthmatics. This review article aimed to describe new approaches to achieve optimal use of the currently available biologics. Interestingly, the authors stated that induced sputum could better predict the response to anti-IL5 biologics, and possibly anti-TSLP. They also highlighted our limited understanding of biomarkers that could predict the outcome of other currently available biologics, or novel biologics that are still under development, thus, underlining the necessity of discovering and validating new biomarkers.

Biologics targeting Th2 inflammation are approved for the treatment of severe chronic rhinosinusitis with nasal polyps (CRSwNP) in addition to their use in severe asthma. The review article by Cottin et al. discusses the potential predictive value of upper airway disease (UAD) diagnosis on the response of patients with severe asthma to biologics. The authors evaluated data emerging from post hoc analyses of randomized, placebo-controlled trials (RCTs) by stratifying a posteriori enrolled patients according to the presence or absence of UAD, specifically allergic rhinitis and/or CRSwNP. The lack of published results from head-to-head comparisons of the use of biologics between severe asthma and CRSwNP led (Cottin et al.) to propose the acronym “ABC”, standing for age/biology/comorbidities, as the three major sets of biomarkers that impact the response to biologics.

Moreover, the article by Gandhi et al. provided interesting insights into the role of Protease-Activated Receptor-2 (PAR_2_) in asthma pathogenesis and its ppotential use as a biomarker. It highlighted the fact that prevention of PAR_2_ activation by allergens or endogenous proteases could result in therapeutic benefit in asthma. The authors also reviewed recent studies on the role of PAR_2_ in allergic asthma and reported that markers of activation of PAR_2_-related pathways could be candidates for biomarkers of asthma severity and/or control.

The article by Specjalski et al. examined whether the chitinase-like protein (CLP) YKL-40 could serve as a biomarker of neutrophilic asthma in everyday practice. Even though an elevated level of serum YKL-40 is strongly associated with neutrophilic and obesity-related phenotypes of asthma, current evidence suggests that its low specificity prevents its application in a clinical setting.

Focusing on asthma phenotyping and endotyping, the original article by Shrestha Palikhe et al. investigated the association of a polymorphism in the CRTh2 gene (rs533116 G > A) with severe asthma and Th2 inflammation in older females (≥45 years). Despite its limitations, this cross-sectional study showed that CRTh2 rs533116 AA genotype is associated with Th2 inflammation and revealed a gene-sex-aging interaction influencing the effect of CRTh2 on asthma severity.

In an opinion article, Domvri and Porpodis, discussed whether there are enough data to support targeting of inflammation vs. remodeling as the best approach for personalized therapy of asthma. They suggest that biologics seem to be the answer in the treatment of severe asthma regarding both inflammation and remodeling based on beneficial results by limited studies so far, that indicate biologic's possible disease modification effect.

We believe that this Research Topic adds to the current literature and advances our understanding of the role of biomarkers in asthma therapy. It is with great pleasure that we are presenting the articles included in this Research Topic to the asthma research community.

## Author contributions

KP: Writing—original draft, Writing—review & editing. IT: Writing—original draft. PS: Writing—review & editing. SG: Writing—review & editing. HV: Writing—review & editing. KD: Writing—original draft, Writing—review & editing.

